# RETRACTED: Skelton et al. Contribution of Host Immune Responses against Influenza D Virus Infection toward Secondary Bacterial Infection in a Mouse Model. *Viruses* 2019, *11*, 994

**DOI:** 10.3390/v16060867

**Published:** 2024-05-29

**Authors:** Raegan M. Skelton, Kelly M. Shepardson, Alexis Hatton, Patrick T. Wilson, Chithra Sreenivasan, Jieshi Yu, Dan Wang, Victor C. Huber, Agnieszka Rynda-Apple

**Affiliations:** 1 Division of Basic Biomedical Sciences, Sanford School of Medicine, University of South Dakota, Vermillion, SD 57069, USA; raegan.nelson@coyotes.usd.edu (R.M.S.); patrick.wilson@coyotes.usd.edu (P.T.W.); 2 Department of Microbiology and Immunology, Montana State University, Bozeman, MT 59717, USA; kelly.shepardson@montana.edu (K.M.S.); alexishatton2222@gmail.com (A.H.); 3 Department of Biology and Microbiology, South Dakota State University, Brookings, SD 57007, USA; chithra.sreenivasan@sdstate.edu (C.S.); jieshi.yu@sdstate.edu (J.Y.); dan.wang@sdstate.edu (D.W.)

The *Viruses* Editorial Office retracts the article, “Contribution of Host Immune Responses Against Influenza D Virus Infection Toward Secondary Bacterial Infection in a Mouse Model” [1], cited above.

Following publication, concerns were brought to the attention of the publisher regarding the accuracy of the images and the image legends for Figures 1, 3B, and 4B, including the failure to incorporate the relevant experimental details that are significant for the interpretation of the findings of this study [1].

Adhering to our procedure, an investigation was conducted by the Editorial Office and Editorial Board, with input provided by the Office of Research Compliance and Montana State University. Following the findings presented within the report provided by the Office of Research Compliance, the failure of the authors to accurately report significant experimental details within Figures 1, 3B, and 4B was confirmed. As a result, the Editorial Board and Editorial Office agreed with the recommendations of the Office of Research Compliance and decided to retract this paper as per MDPI’s retraction policy (https://www.mdpi.com/ethics#_bookmark30) and in line with the Committee on Publication Ethics’ retraction guidelines (https://publicationethics.org/retraction-guidelines).

This retraction was approved by the Editor-in-Chief of the journal *Viruses*.

The authors Raegan M. Skelton, Kelly M. Shepardson and Agnieszka Rynda-Apple agreed to this retraction. The remaining authors did not provide a comment on this decision.
